# Large soluble CD18 complexes with exclusive ICAM-1-binding properties are shed during immune cell migration in inflammation

**DOI:** 10.1016/j.jtauto.2025.100266

**Published:** 2025-01-05

**Authors:** Alexey Ferapontov, Anders Mellemkjær, Helen M. McGettrick, Thomas Vorup-Jensen, Tue W. Kragstrup, Kristian Juul-Madsen

**Affiliations:** a Department of Biomedicine, Aarhus University, Denmark; b Rheumatology Research Group, Department of Inflammation and Ageing, University of Birmingham, Birmingham, B15 2TT, UK; c Rheumatology Section, Diagnostic Center and University Clinic, Silkeborg, Denmark

**Keywords:** Rheumatoid arthritis, Biomarker, Integrins, Soluble CD18, ICAM-1, CD11, MCP-1

## Abstract

The family of heterodimeric CD11/CD18 integrins facilitate leukocyte adhesion and migration in a wide range of normal physiologic responses, as well as in the pathology of inflammatory diseases. Soluble CD18 (sCD18) is found mainly in complexes with hydrodynamic radii of 5 and 7.2 nm, suggesting a compositional difference. Earlier work reported that the complexes include at least part of the CD11a or CD11b chains containing the intercellular adhesion molecule (ICAM)-1 binding domain, and that sCD18 is capable of quantitatively competing with the cell membrane-bound form for ICAM-1 binding. However, it is not clear if the size differences between the sCD18 complexes reflect any functional variance regarding shedding from the cell membrane or binding to ICAM-1. Here, we show evidence that sCD18 found in serum regulates release of the proinflammatory cytokine monocyte chemoattractant protein-1 (MCP-1/CCL2) from fibroblast-like synovial cells. Further, only large sCD18 complexes are capable of binding to ICAM-1. Migrating neutrophils shed large, but not small, sCD18 complexes. Together, these observations explain results measured from patients with rheumatoid arthritis (RA), where large sCD18 complexes dominated in local inflammatory processes involving neutrophil influx into zones of inflammation. Our data points to a previously unappreciated aspect of sCD18 integrin biology as regulators of inflammation in the context of migrating leukocyte. Surprisingly, this regulation is tied to sCD18 complex size, opening new opportunities for therapeutic intervention in serious inflammatory diseases such as arthritis.

## Highlights

• We have developed a novel assay for assessment of soluble CD18 complex size.
• Only large soluble CD18 molecules bind ICAM-1.
• Only large soluble CD18 molecules are shed during neutrophil migration.
• Soluble CD18 complex size shows promise as a biomarker in rheumatoid arthritis.

## 1 Introduction

CD18 integrins are heterodimeric integrins receptors, predominantly expressed in the cell membrane of most leukocyte subsets [1]. In the membrane a non-covalent complex is made between either of the CD11a, CD11b, CD11c, or CD11d–150 kDa alpha-chain carrying the ligand binding domain, and the ∼95 kDa CD18 beta-chain. These receptors are fundamental in cellular adhesion and diapedesis through the endothelium and with importance for phagocytic anti-microbial defense mechanisms and immune-mediated inflammatory diseases [1,2]. However, several reports have now challenged the classic description of the CD18 integrins as only a part of the membrane environment [[3], [4], [5], [6]]. Evans et al. identified soluble forms of (s)CD18 in artificially induced blisters, leaving behind a stub of CD18 in the cell membrane of extravasated neutrophils [3]. Gjelstrup et al. reported sCD18 complexes in blood from normal donors and leukocyte culture supernatants [4], suggesting these complexes as part of normal physiology. Shedding of CD18 can occur in vitro following stimulation of neutrophils or macrophages with pro-inflammatory cytokines, such as tumor necrosis factor alpha (TNF-α), or upon cell migration [4,7,8]. More detailed analyses identified a group of matrix metalloproteinases and serprocins as the likely sheddases, cleaving both alpha and beta chains of the CD18 integrins [[7], [8], [9]]. From gel permeation chromatography (GPC) analyses, Gjelstrup et al. reported that sCD18 in blood from healthy individuals are found as distinct complexes, mainly with hydrodynamic radii (*R*_H_) of 5 and 7.2 nm. However, our understanding of the formation and function of these complexes remains limited.

sCD18 can bind intercellular adhesion molecule (ICAM)-1 and the complement fragment iC3b. Analyses with enzyme-linked immunosorbent assay (ELISA)-like methods suggest that complexes between sCD18 and soluble forms of CD11a or CD11b exist in both mice and human, while similar complexes with CD11c or CD11d have not been reported [5,10]. Since the ligand for CD11a is ICAM-1 and for CD11b are ICAM-1 and iC3b, their inclusion in the sCD18 complexes matches the known ligand binding properties [2]. Several in vitro experiments have shown that the binding of sCD18 to ICAM-1 competes with the binding of membrane bound CD18 to ICAM-1 [5]. Translated into a physiologic setting, such a capacity may function as an anti-inflammatory molecule limiting the ability of endothelial to support the adhesion of circulating leucocytes [[4], [5], [6]]. In this context, the observation made by Gjelstrup et al. of a polydisperse size distribution of the sCD18 complexes is of interest as potential differences in the way these complexes interact with the endothelium could impact their relative distribution, but such information remains unknown.

The concentration of sCD18 complexes has previously shown alterations both up and down compared to healthy controls in immune-mediated inflammatory diseases (IMID), including rheumatoid arthritis (RA) and spondyloarthritis (SpA), as well in diseases with a strong activation of the immune system such as sepsis and acute alcoholic hepatitis [5,6,11,12]. These findings suggest that sCD18 could be a part of the pathology of immune activation, but the mechanistic insight is ambiguous. Both abnormally high and low concentrations of sCD18 have been associated with poor disease progression in sepsis [6]. However, these measurements did not differentiate between the large and smaller complexes of sCD18 [6]. One may suggest the complexes reflect molecular characteristics of the inflammatory response. For instance, Gjelstrup reported observational evidence that patients with RA and SpA differed in their sCD18 size profiles with RA patients having more large sCD18 molecules, but with no explanation for the source of these differences [13]. Also supporting the view of large protein complexes as potential indicators of inflammatory activity, large protein complexes of proteins other than CD18 were reported to form in blood and synovial fluid (SF) from systemic lupus erythematosus (SLE) and RA patients, respectively [[14], [15], [16]] with clear connection to the level of pathological inflammation. Therefore, the full biomarker potential of sCD18 is still unresolved. The combined information on concentration and structure of such protein aggregates could strengthen biomarker value.

In this study, we aimed to characterize the oligomeric structure and function of sCD18 shed during inflammatory processes. Next, we analyzed the contribution of different structural populations of sCD18 to one of its most prominent biological functions namely binding of ICAM-1. Lastly, we wanted to investigate how any differences in sCD18 structure would affect disease activity in RA and how large sCD18 could be a predictive marker in autoinflammatory diseases.

We now report that sCD18 suppresses secretion of monocyte chemoattractant protein (MCP)-1 by the ICAM-1 expressing fibroblast-like synovial cells (FLS). While previous reports found that sCD18 binds ICAM-1, our analysis assigns this activity only to the larger sCD18 complexes. Likewise, we find that sCD18 complexes shed from migrating neutrophils are of the large-size type. Finally, mirroring the in vitro experiments, these large sCD18 complexes are also related to local inflammatory processes seen in RA where higher concentrations of large sCD18 complexes are found in both plasma and SF. Our study opens new perspectives on ultrastructural changes in soluble protein complexes as part of normal immune regulation versus the pathological consequences of IMID.

## 2 Materials and methods

### 2.1 Patients and healthy controls

Plasma and SF from patients with RA with a disease flare were collected at Department of Rheumatology, Aarhus University Hospital for the INflammation in ARThritis (INART) biobank (n = 35) (Table 1) [6]. All patients included in this study were >17 years of age fulfilling the EULAR/ACR 2010 classification criteria for RA [17]. All samples were collected in EDTA tubes and stored at −80 °C until analysis. Synovial fluid mononuclear cells (SFMCs) and peripheral blood mononuclear cells (PBMC) were isolated by conventional Ficoll-Paque (GE Healthcare) density-gradient centrifugation and cryopreserved at −135 °C. FLS were grown from SFMCs as previously described [[18], [19], [20]]. Plasma and serum samples from healthy controls, were obtained from the Danish Blood Bank, Aarhus University Hospital. Specific information on the mean age and sex of healthy controls was not released from the Blood Bank. Danish blood donors are aged between 18 and 60 years.

**Table 1:** Patient characteristics.

Chronic RA patients (n = 35)	
**Age (years)**	58 (41–66)
**Gender (% female)**	64
**Treatment**	
**csDMARD (%)**	63
**NSAID (%)**	3
**bDMARD (%)**	26
**Disease activity**	
**CRP (mg/mL)**	8 (2.6–26)
**Patient global (1**–**100)**	26.5 (49–76.5)
**Patient pain (1**–**100)**	47 (22–71.5)
**Physician global (1**–**100)**	31 (14–43)
**HAQ (0**–**3)**	0.75 (0.075–1.75)
**DAS28CRP (0**–**10)**	3.57 (2.97–4.99)

Table 1: Data are expressed as median with IQR. csDMARD, conventional synthetic disease modifying anti-rheumatoid drug. bDMARD, biologic disease modifying anti-rheumatoid drug. NSAID, non-steroid anti-inflammatory drug. CRP, C-reactive protein. HAQ, health assessment questionnaire. DAS28CRP, disease activity score 28 based on C-reactive protein.

### 2.2 Ethics

All human samples were obtained after informed written consent according to the Declaration of Helsinki. The Danish Data Protection Agency and the Ethics Committee at Region Midt approved the collection of SF and peripheral blood (20121329). Alternatively, approval from the Human Biomaterial Resource Centre (Birmingham, UK), Northeast Tyne and Wear South Research Ethics Committee (15-NE-0285) or University of Birmingham Local Ethical Review Committee (ERN_12–0079) was provided for the collection of human umbilical vein endothelial cells (HUVEC) and human neutrophils from healthy donors.

### 2.3 sCD18-depletion studies in vitro

sCD18 was depleted from normal human serum or from the supernatant of SFMCs 1 × 10^6^ cells/ml cultured at 37 °C and 5 % CO2 for 48h in RPMI medium supplemented with 10 % fetal calf serum (FCS), penicillin (100 units/mL), streptomycin (100 μg/mL) and glutamine (2 mM) at [6]. Briefly, 48-well culture plates were coated with either mouse IgG1 antibody to human CD18 (KIM18; GenScript, Piscataway, NJ) or isotype IgG1 (X093101-2, Agilient, Santa Clara, CA) both 5 μg/ml in 500 μl PBS for 24h at room temperature (RT). Wells were emptied and washed in PBS. Then, 500 μl of normal human serum (NHS) or SFMC supernatant was added to the wells for 2h, followed by washing in 500 μl PBS and repeated twice. The sCD18-depleted NHS or SFMC supernatant was then harvested, aliquoted, and frozen at −80 °C.

Co-cultures were made with FLS and PBMC. FLS from RA patients were resuspended in a concentration of 4 × 10^4^ cells/ml in DMEM (Gibco, Cat. A4192102) with 10 % FCS, penicillin (100 units/mL), and streptomycin (100 μg/mL) and grown at 37 °C and 5 % CO_2_ in 96-well plates for 24h with 10,000 cells/well. The next day, healthy donor PBMC (1 × 10^6^ cells/ml) were resuspended in RPMI medium (Gibco, Cat. 11875093), supplemented with 10 % FCS, penicillin, streptomycin and glutamine as above. The medium was removed from FLS cultures and 1 × 10^5^ PBMC were added per well to start the co-culture. Monocultures of FLS or PBMC were included as controls. NHS and SFMC culture supernatants, with and without depletion of sCD18, were added and cultures incubated for 48h at 37 °C and 5 % CO_2_. Finally, supernatants were harvested, centrifuged at 100,000×*g*, and frozen at −80 °C until analysis by a commercial MCP-1 ELISA kit (R&D Systems, Cat. DCP00, Minneapolis, MN, USA).

### 2.4 MCP-1 ELISA

MCP-1 levels in the supernatant were quantified using a commercially available MCP-1 ELISA kit (R&D Systems, Minneapolis, MN, USA) according to the manufacturer's instructions. Briefly, 96-well plates pre-coated with a monoclonal antibody specific for human MCP-1 were used. Standards, controls, and samples were added, a biotinylated detection antibody specific for MCP-1 was added, followed by streptavidin-HRP and tetramethylbenzidine (TMB) buffer. The reaction was stopped, and absorbance was measured at 450 nm with a correction reading at 570 nm to subtract background absorbance. All samples were analyzed in duplicate. A standard curve was generated using serial dilutions of known concentrations of recombinant human MCP-1, and sample concentrations were calculated by interpolating absorbance values against the standard curve. All data points were verified to ensure they fell within the linear range of the standard curve and plotted in pg/ml or as a ratio compared to values from a FLS monoculture.

### 2.5 GPC fractionation of plasma and culture supernatants

HC plasma, 6 INART patient plasma samples, and supernatants from the transwell migration assay described below were analyzed by GPC on a HiLoad™ 16/60 Superdex™ 200 column with a bead volume of 120 ml, operated in an ÄKTA FPLC™ chromatography system (Pharmacia AB, Uppsala, Sweden). Samples of 500 μl or 1 mL were centrifuged at 10,000×*g* and injected with 140 mM NaCl, 10 mM Tris-HCl, pH 7.4 (TBS) with 0.05 % (v/v) Tween-20 (Tw) and 0.099 % (w/v) NaN_3_ (azide) as running buffer and a flowrate of 1 ml/min 120 fractions of 1 ml were collected for each sample. sCD18 levels were measured in fractions by time-resolved immunofluorometric assay (TRIFMA) as described below. *R*_H_s for the sCD18 was estimated as described earlier [4] from a set of calibration markers with known *R*_H_, including thyroglobulin (*R*_*H*_ = 8.5 nm), ferritin (*R*_*H*_ = 6.1 nm), aldolase (*R*_*H*_ = 4.8 nm), conalbumin (*R*_*H*_ = 3.6 nm), and ovalbumin (*R*_*H*_ = 3.1 nm).

### 2.6 Quantification of sCD18 by time-resolved immunofluorometric assay

sCD18 in plasma samples, SF samples, or cell supernatants was detected by TRIFMA as described earlier [4]. Briefly, Maxisorp™ microtiter wells (437958, FluroNunc™, Thermofisher, Waltham, MA) were coated for 24h at 4 °C in a humid chamber with mAbs to human CD18 (KIM185 or TS1/18) or an isotypic control murine IgG1κ (Dako, M7894) at 1 μg/ml in 100 ml PBS. Residual binding sites were blocked with 200 ml TBS, 1 mg/ml HSA for 1h at room temperature (RT). Heat-aggregated IgG (ΔIgG) was prepared by incubating human IgG (Beriglobin™, 007815; ZLB Behring, Hattersheim am Main, Germany) at 56 °C for 30min to create irreversible IgG aggregates suitable for inclusion in the assay buffer [21,22], followed by centrifugation at 13,000×*g*. Wells were washed 3 times in TBS/Tw/azide. Samples were then diluted in TBS/Tw/azide with 1 mM CaCl_2_, 1 mM MgCl_2_, and 100 μg/ml ΔIgG added, incubated for 24h at 4 °C in wells with 100 μl of the sample dilution, and then washed as described above. Next, wells were incubated for 1h at RT with 100 ml biotinylated mAbs against CD18 (KIM127, TS1/18) [4], diluted to 1 μg/ml in TBS/Tw/azide with 1 mM CaCl_2_, 1 mM MgCl_2_, and 100 μg/ml bovine IgG (001-000-003, Jackson ImmunoResearch, West Grove, PA). After three washes, 100 μl of Eu^3+^-conjugated streptavidin (PerkinElmer), diluted 1000 times in TBS/azide with 25 μM EDTA, was added to the wells and followed by 1h incubation at RT. Lastly, the wells were washed, and the signal was read by time-resolved fluorometry (Victor 3™ 1420 multi label counter). The signals from samples and supernatants were compared to a standard curve made from a titration of HC plasma defined to contain 1000mU/ml sCD18 [4]. For inter-assay control, 2 HC plasmas were repeatedly applied on each 96-well plate.

### 2.7 Surface plasmon resonance-based assays

Preparation of CM4 sensor chips (GE Healthcare, Little Chalfont, UK) and recording of sensorgrams was carried out in a BIAcore 3000 instrument (GE Healthcare). Immobilization of iC3b (CompTech, Cat. A115) and ICAM-1 (R&D Systems, Cat. ADP4), was done through amine coupling as described previously. As a control surface, an activated-and-blocked chip surface with no protein was also prepared [23].

50 μl of GPC-fractionated plasma samples, corresponding to either the peak in sCD18 concentration at *R*_H_ = 7.2 nm (large complexes) or *R*_H_ = 5 nm (small complexes), were mixed with 50 μl of either *i)* TBS/Tween/azid with 2 mM MnCl_2_ and 0.2 mM CaCl_2_ to activate integrins for ligand binding, *ii)* TBS/Tween/azid with 2 mM MgCl_2_ and 0.2 mM CaCl_2_ to permit ligand binding of pre-activated integrin, or *iii)* TBS/Tween/azid with 10 mM EDTA to block integrin ligand binding [24]. To avoid a post-injection set-off in sensor signal due to alterations in divalent cation concentrations, the running buffer for the experiments were carefully adjusted to match the diluted samples such that these contained i) TBS/Tween/azid/1 mM MnCl_2_/0.1 mM CaCl_2_, ii) TBS/Tween/azid/1 mM MgCl_2_/0.1 mM CaCl_2_, or iii) TBS/Tween/azid/5 mM EDTA, respectively. The diluted samples were injected in-line over the control and ligand-coupled surfaces with a flow rate of 5 μl/min and a contact time of 480s, followed by a dissociation phase of 120s. The sensor chips were regenerated in 1.5M NaCl_2_, 50 mM EDTA, 100 mM HEPES (pH 7.4).

The recorded sensorgrams were aligned within the BIAevaluation software (GE Healthcare) and the signals from control surfaces was subtracted paired signals from the ligand-coated cells. The binding was analyzed using EVILFIT version 1.2 software [25], extracting the distribution in binding kinetics from the sensorgrams as described before [26,27]. Following the estimates from TRIFMA analysis, the applied concentration of sCD18 was loaded into the algorithm in milliunits from comparison with the standard serum. As previously, the extracted ensemble of 1:1 reactions were plotted in 2D diagrams typifying each 1:1 reaction by the pseudo-K_D_ (in standard units) on the abscissa, the dissociation rate (*k*_d_, in s^−1^) on the ordinate, and the abundance of the reaction indicated with color contours (in resonance units [RU]).

### 2.8 Isolation and culture of endothelial cells

Human umbilical vein endothelial cells (EC) were isolated from umbilical cords as previously described [28,29] and cultured in Medium 199 (Life Technologies, Paisley, UK) supplemented with 20 % FCS, 35 μg/ml gentamicin, 10 ng/ml epidermal growth factor, 1 μg/ml hydrocortisone (all from Sigma-Aldrich, Paisley, UK) and 2.5 μg/ml Amphotericin B (Life Technologies). EC were dissociated using trypsin/EDTA (Sigma) and seeded on either tissue culture plates (Falcon; Becton Dickinson Labware, NJ, USA) or uncoated low-density 3.0 μm pore polycarbonate Transwell™ filters (BD Pharmingen, Oxford, UK) placed in matching plates at a cell density to yield confluent monolayers within 24h [28,30,31]. Confluent monolayers were treated with 0 or 100U/ml TNFα (R&D Systems, Abingdon, UK) for 4h or 24h at 37 °C in a CO_2_ incubator before the assay [30,32,33].

### 2.9 Isolation of neutrophils

Venous blood was collected from healthy donors into EDTA tubes (Sarstedt, Leicester, UK). Neutrophils (PMN) were isolated by centrifugation on two-step histopaque density gradients as previously described [32,33]. Purified neutrophils were washed twice in M199 with 0.15 % bovine serum albumin (M199BSA; Sigma) with centrifugation at 250×*g* for 5min, counted, and re-suspended to the desired final concentration in M199BSA.

### 2.10 Neutrophil migration assay

EC in 3 μm pore Transwell filter inserts were transferred to fresh plates and washed twice in M199BSA to remove residual TNFα. Purified neutrophils (400 μl at 2 × 10^6^ cells/ml) were added to the upper chamber of 12-well filter, and allowed to settle at 37 °C in a CO_2_ incubator for 4h or 24h. Supernatants were collected from the upper (non-migrated cells) and lower (migrated cells) chambers, centrifuged at 250×*g* for 5min and stored at −80 °C until analysis. To assess the effects of direct neutrophil-EC interactions, neutrophils (1 × 10^6^ cells/ml) were allowed to adhere directly to the EC monolayer (direct contact) for either 4h or 24h. Supernatants were collected, centrifuged at 250×*g* for 5min and stored at −80 °C until analysis of sCD18 concentration and complex size.

### 2.11 Statistical analysis

Patient characteristics were described by the median and interquartile range (IQR). Normality of data was assessed using QQ-plots. Data that was not normally distributed was analyzed using non-parametric statistical tests. Concentrations of sCD18 were analyzed with the Wilcoxon signed rank test for paired data and the Mann-Whitney *U* test for unpaired data. Ratios of sCD18 concentrations were analyzed with the paired *t*-test for paired data and the *t*-test for unpaired data. A two-tailed p-value below 0.05 was considered significant. Statistical calculations and graphs were made using GraphPad Prism version 6 (GraphPad Software, San Diego, CA).

## 3 Results

### 3.1 sCD18 complexes dampen immune response in vitro

**Fig. 1:**
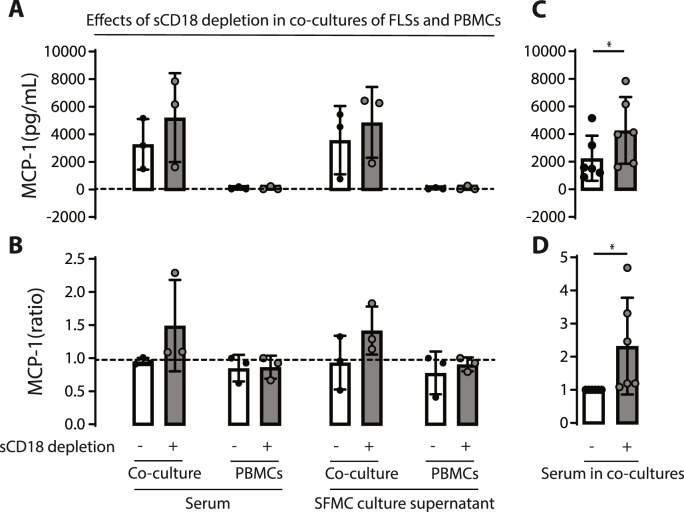
A) Effect of sCD18 depleted (grey bars) serum and supernatant compared to non-depleted serum and supernatant (white bars) on MCP-1 secretion by co-cultures of FLS and PBMC (n = 3 donors) or monocultures of PBMC (n = 3). Dashed lines represent levels in a monoculture of FLS used for standardization (n = 1). Data is plotted as pg/ml in accordance with standard curve calculated using manufacturer provided materials. Depletion of sCD18 resulted in increased secretion of MCP-1 in co-cultures of FLS and PBMC. B) Change in MCP-1 ratio compared to FLS monoculture. C) Effect of sCD18 depleted serum on MCP-1 secretion by co-cultures of FLS and PBMC (n = 6 with 2 different FLS donors and 6 different PBMC donors). D) Change in co-culture ratio of MCP-1 production as a result of sCD18 serum depletion. Bar graphs represent mean values and error bars represent SEM. Difference between MCP-1 concentration dependent on sCD18 depletion in C) was tested using a Wilcoxon signed rank test and in D) paired *t*-test. ∗ marks p < 0.05.

To investigate the possible anti-inflammatory properties of sCD18, we used co-cultures of FLS and PBMC to simulate the interaction of stromal cells and leukocytes in inflammation. First, monocultures of both PBMC (n = 3) and FLS (n = 1) and co-cultures of FLS and PBMC (n = 3) were incubated with serum or supernatant without or with specific depletion of sCD18 and assessed for MCP-1 production (Fig. 1). sCD18 levels were successfully depleted from 727 mU/ml in NHS containing isotype IgG1 compared to 314 mU/ml in NHS with KIM185 antibody. sCD18 levels in SFMC supernatant decreased from 122 mU/ml for isotype IgG1 controls to 53 mU/ml in KIM185 antibody treated samples. The production of MCP-1 tended to be increased in the co-cultures treated with sCD18-depleted serum or supernatants compared to sCD18 containing serum or supernatants (Fig. 1A–B). The production of MCP-1 in monocultures of both PBMC and FLS was barely detectable, with no differences when comparing cultures with or without sCD18 depletion (Fig. 1A). Subsequently, co-cultures of FLS and PBMC (n = 6, 2 FLS donor and 6 PBMC donors) were incubated with serum with or without depletion of sCD18 and assessed for MCP-1 production. In these experiments, the production of MCP-1 was significantly increased in the co-cultures treated with sCD18-depleted serum compared with sCD18 containing serum (Fig. 1C–D).

### 3.2 2D kinetic analysis of sCD18 binding show large but not small sCD18 complexes facilitate ICAM-1 binding

**Fig. 2:**
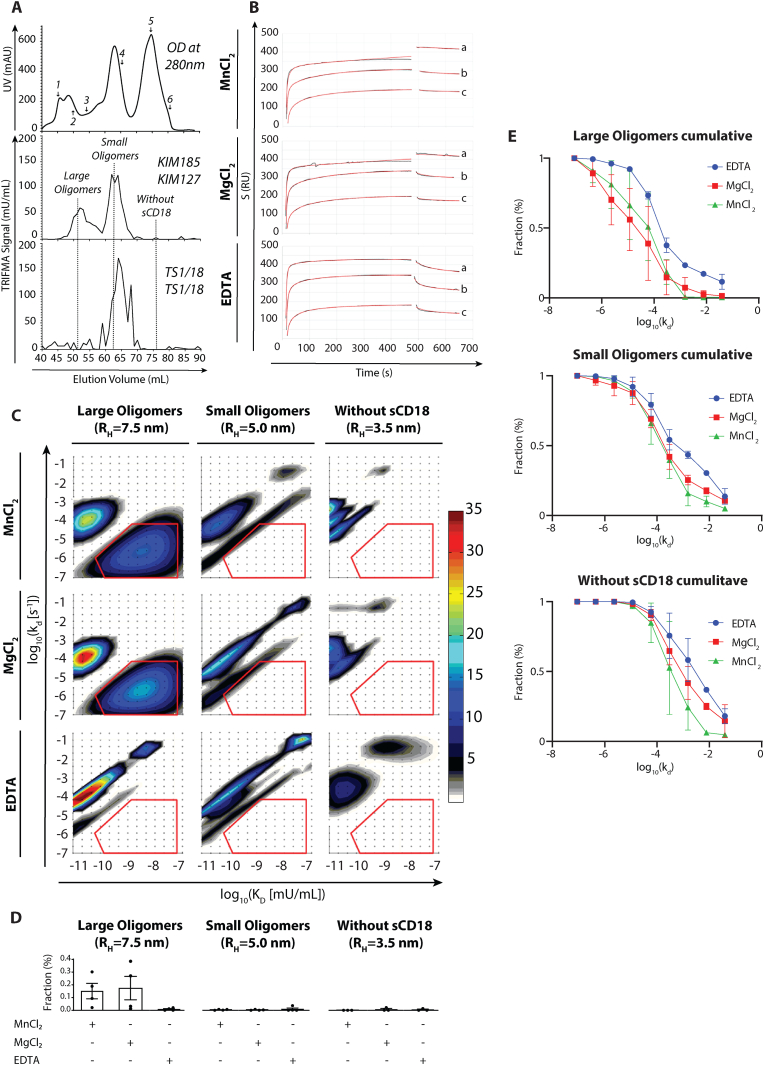
A) Average chromatogram of plasma fractionation using GPC. Protein in each fraction was measured by UV absorbance at 280 nm. Fractions were then analyzed by TRIFMA in two assays, namely Assay 1 with KIM185 (coat.) KIM127 (detect.) or Assay 2 with TS1/18 (coat.) and TS1/18 (detect.) (n = 4). B) SPR analysis of binding interactions between analytes of large sCD18 faction (a), small sCD18 fraction (b), or a fraction with no sCD18 and ICAM-1. Dilution and running buffer contained MnCl_2_, MgCl_2_, or EDTA. The SPR signal was plotted in RU as a function of time (black curves). The EVILFIT software was used to fit the experimental curves (red curves) (n = 4). C) 2D-binding kinetic analysis based on the fitted sensorgrams in B). Binding populations are separated based on *log*_10_*K*_D_ values (X-axis) and *log*_10_*k*_d_ values (Y-axis). The abundance of each binding type is indicated in RU with a color gradient. The BIN1 area (red box), quantified interactions unique to conditions permitting integrin binding. D) Quantification of BIN1 binding population in each experiment was normalized against the amount ICAM-1 coated onto SPR chip for each individual experiment. The resulting values were plotted as mean values for either three or four experiments, with standard error of mean (n = 3–4). E) Normalized cumulative distribution of dissociation rate constants (*log*_10_*k*_d_) derived from the 2D plots, enabling direct comparison of the distribution of fast and slow interacting complexes with ICAM-1.

As also shown in the past [4], TRIFMA results indicate that normal human plasma contains sCD18 integrin complexes mainly found in distributions around two different hydrodynamic radii (Fig. 2A). No information has been available on their ability to bind ligand.

We made use of an SPR-based assay with ICAM-1 as a ligand and GPC-fractions enriched in either of the two types of sCD18 complexes injected as analyte. As control we used a protein-containing GPC fraction with no detectable sCD18. To further distinguish integrin binding from unspecific interaction with chip surface, we used three metal-ion environments, namely a buffer with Mg^2+^ or Ca^2+^ permitting binding of already activated integrin, and EDTA-containing buffer, which abolishes integrin binding. We applied the samples with minimal dilution and recorded a single sensorgram for each condition in each experiment. The binding kinetics information was then extracted from the sensorgram by use of an algorithm permitting the characterization of heterogeneous interactions between analytes and ligand [25] from a single sensorgram as validated previously [26]. This use was intended to identify, at least partly, non-integrin related interactions from binding both in the presence and absence of EDTA and maybe as well in the non-sCD118 containing fraction. In contrast, the sCD18 signal of interest was expected to show up in the divalent cation-containing buffers, strengthened by the presence of Mn^2+^ compared to Mg^2+^.

The sensorgrams showed a clear separation between fractions containing different sCD18 oligomers for all chosen buffers (Fig. 2B). Binding signals followed a logical order of large sCD18 oligomers displaying the highest signal, and therefore bind strongest to the chip surface, followed by the small sCD18 oligomers, and the fraction with no sCD18 oligomers having weakest binding (Fig. 2B).

The SPR analysis calculates the minimal ensemble of 1:1 binding reactions required to account for the experimentally measured binding of soluble injectant (analyte) to ligands immobilized on the chip surface. In our experiment, no information was available on the molar concentration of sCD18 complexes as their precise molecular weight is not known, nor is the conversion of units into g/L. In this situation, we chose to use the concentration in mU/mL as a pseudo-concentration. Each 1:1 binding reaction is typified by paired association (*k*_d_) and association (*k*_a_) rates. Results are shown as a three-dimensional plot, where *x* and *y* coordinates indicate the pseudo-*K*_D_ (in mU) derived from *K*_D_*=k*_d_*/k*_a_, and k_*d*_ (s^−1^), respectively, and z coordinate indicates the binding signal (in RU) (Fig. 2C). While the pseudo-*K*_D_ is not a true thermodynamical measure, it still helps to indicate the concentration of sCD18 required to quantitatively saturate interactions from the Langmuir formula, i.e., with 50 % saturation at *c* = *K*_D_ (both in mU).

To capture relevant binding populations, the algorithm was set to probe interactions on a grid defined as an interval of 4.5^−12^ mU < *K*_*D*_ < 10^−7^ mU and an interval of 9^−8^ s^−1^< *k*_*d*_ < 0.2 s^−1^ for all analyzed experiments (Fig. 2C). The 2D analysis of binding permitted an interpretation following the scheme mentioned above. The contour plots revealed interactions found under all experimental conditions, and others, which were unique to a specific fraction or buffer. Top right corner population, with *K*_D_ *≈* 10^−8^ mU and *k*_d_ *≈* 10^−1.5^ s^−1^ was recurring for several fractions and buffers. The relatively high *k*_d_ value suggested unspecific, weak binding with fast dissociation rates such as an unspecific interaction with plasma components not related to sCD18 integrins. Contrary, a binding population in bottom right (BIN1), with *K*_*D*_ *≈* 10^−8.5^ mU and *k*_*d*_ *≈* 10^−6^ s^−1^ (indicated with red polygon), was unique for fractions containing large sCD18 oligomers. Furthermore, it was absent in EDTA buffer, suggesting requirement of divalent metal ions for these binding interactions. The combination of a relatively low *k*_*d*_ value, specific to fractions with large sCD18 oligomers, and requiring buffers with divalent metal ions, suggested that this binding population was generated by integrin binding of ICAM-1. BIN1 was quantified across individual SPR experiments (n = 3–4) (Fig. 2D). As it was visualized in 3D plots (Fig. 2C), the mean value from independent SPR experiments repeated that BIN1 signal is unique for fractions containing large sCD18 oligomers that only occurs in the presence of divalent ions, Mn^2+^ and Mg^2+^. The remaining fractions, as well as negative buffer controls showed almost complete absence of this type of binding interaction, based on the mean signal strength (Fig. 2D).

While the estimation *K*_D_s suffers from the lack of a proper molar concentration for the sCD18 complexes, according to the Langmuir binding scheme the dissociation phase signal follows a simple exponential function and is independent of the analyte (sCD18) concentration. Consequently, the calculated *k*_d_s are proper thermodynamic dissociation rates. We analyzed the total distribution of binding populations across the full range of *k*_*d*_ values as previously done for other large oligomers [27]. The fractions containing large sCD18 oligomers were dominated by slow dissociation rate binding populations in the presence of divalent cations. Fractions containing small and no sCD18 complexes, displayed distributions comparable to EDTA containing conditions, arguing for a weak or no contribution to binding (Fig. 2E). Altogether, these binding kinetics indicate that only large sCD18 complexes are quantitatively responsible for the interaction with ICAM-1. Furthermore, this interaction was dependent on divalent cations such as Mg^2+^ or Mn^2+^ in agreement with the expectations for integrin binding.

### 3.3 Migrating neutrophils shed large sCD18 complexes at the inflammatory site

**Fig. 3:**
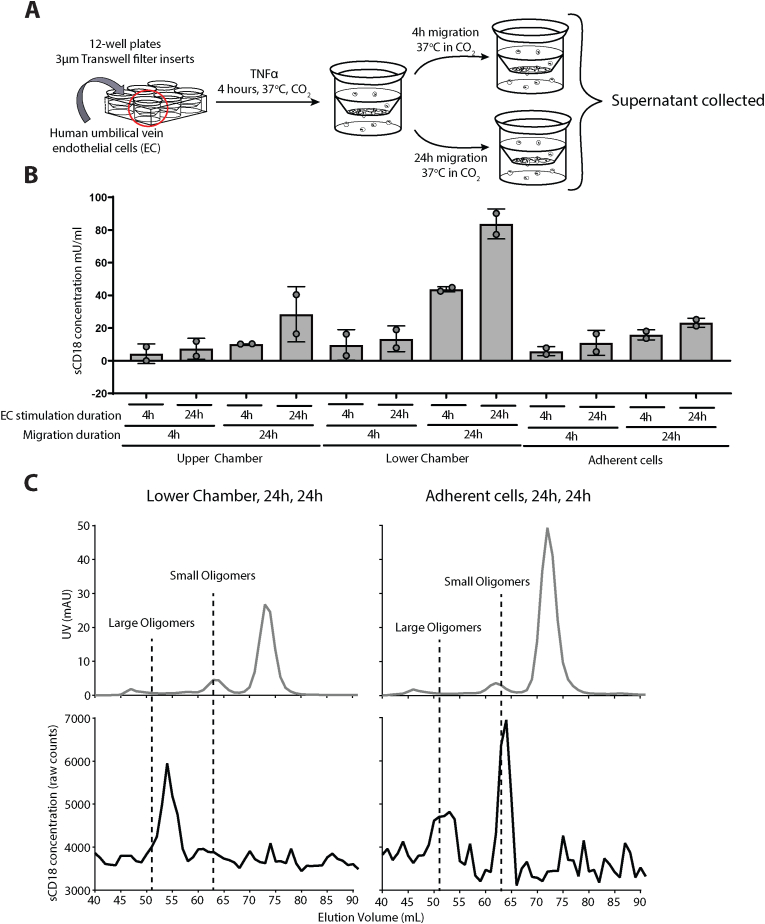
A) Schematic illustration of neutrophil migration assay. Supernatants were collected from the non-migrated cells (upper chamber) and migrated cells (lower chamber). B) The concentrations of sCD18 measured in supernatants from upper chamber, lower chamber, adherent cells, and monocultures of endothelial cells (ECs) (all n = 2). Data is plotted as mU/ml compared to internal standard plasma. Endothelial cells were pre-incubated with TNFα (EC stimulation duration) for either 4 h (4h) or 24 h (24h). Neutrophils were allowed to migrate (migration duration) for either 4 h (4h) or 24 h (24h). C) sCD18 concentration (black, lower row) and absorbance (grey, uooer row) measured in 1 ml fractions of supernatants from migrated cells (lower chamber) and neutrophils added to endothelial cell monolayer (adherent cells). Absorbance is a measure of total protein level. sCD18 concentration is plotted as raw TRIFMA counts. Boxes and error bars indicate median and interquartile range.

**Fig. 4:**
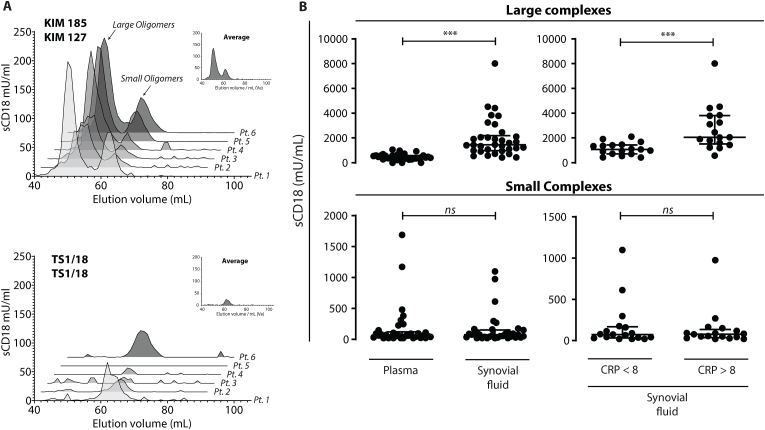
Concentration of large and small sCD18 complexes in plasma and SF from patients with RA. A) Individual and averaged measurements of sCD18 concentrations measured as mU/ml in the elution fractions by two TRIFMA assays, KIM185/KIM127 (Assay 1) and TS1-18/TS1-18 (Assay 2), in plasma of 6 rheumatoid arthritis patients. B) The concentration of large and small sCD18 complexes measured by assay 1 and 2 in mU/ml in plasma and SF from patients with RA (Left Side). The concentration of large and small sCD18 complexes in SF from patients with RA with elevated CRP levels (CRP >8 mg/l) or normal CRP levels (CRP <8 mg/l) (Right Side). Data was tested using Wilcoxon (left side) and Mann Whitney t-tests (right side). ∗∗∗ marks p < 0.001.

Next, we investigated the timing of CD18 shedding during migration of primary leukocytes and the sizes of the resulting sCD18 complexes using a filter-based experiment. Supernatant from adherent, but non-migrated, neutrophils and from neutrophils after transendothelial migration through the endothelial cell layer were collected after 4 and 24 h (Fig. 3A). As expected, no sCD18 was detected in the supernatants from EC monocultures (Fig. 4B). sCD18 levels tended to increase in supernatants from migrated neutrophils (lower chamber) following 24h of migration compared to 4h or in supernatants from non-migrated cells (upper chamber) and adherent cell conditions (Fig. 3B). Moreover, higher levels of sCD18 concentration were seen following EC stimulation for 24h compared to 4h (Fig. 3B). Shedding of sCD18 required both a prolonged interaction with ligands on the surface of ECs, as well as full migration across the endothelial barrier.

Analysis of the size distribution of sCD18 shed during migration of leukocytes was made with GPC as for plasma. The supernatant from the migrated neutrophils (lower chamber) was passed through a molecular mass calibrated Superdex200 column (Supp. Fig. 1) and collected in fractions of 1 ml, and sCD18 in the fractions was measured using TRIFMA. Supernatant from neutrophils on endothelial cell monolayer (adherent cells) was used as a control (Fig. 3C). Strikingly, the supernatant from migrated neutrophils (lower chamber) contained predominantly large sCD18 complexes with *R*_H_ of 7.2 nm. In contrast, the supernatant from neutrophils on endothelial cell monolayer (adherent cells) contained mainly small complexes with *R*_H_ of 5 nm and to a lesser extent large complexes similar to those generated by the migrated neutrophils.

### 3.4 Large sCD18 complexes dominate synovial fluid from inflamed patients

As gel filtration is time-consuming and not easily implemented with testing of many samples, we wanted to establish an alternative method for detecting small and large sCD18 complexes. Plasma from 6 RA patients was passed through the GPC column. The sCD18 was measured in all fractions using two different TRIFMA assays (supp. Fig. 2). These TRIFMA assays differ in the way that Assay 1 used two different monoclonals (KIM127 and KIM185) to CD18 as capture and detection antibodies while Assay 2 used the same monoclonal antibody (TS1/18) for both capture and detection. Assay 1 generated a signal both at *V*_e_ ranging from 60 to 67 ml (small complexes) and at *V*_e_ ranging from 48 to 57 ml (large complexes). Assay 2 only generated a significant signal at *V*_e_ ranging from 60 to 67 ml (small complexes) overlapping the elution volumes for small complexes detected by Assay 1. A subtraction of the signal from Assay 2 from the signal of Assay 1 was used to make estimations of large sCD18 complex concentrations, while the signal from Assay 2 alone was used to make estimations of the small sCD18 complex concentrations (Fig. 4A). With this procedure, we studied sCD18 complexes in both peripheral plasma and at the inflammatory site with SF samples from RA patients. Plasma represents the compartment from where leukocytes exit the blood to enter the tissue, and SF represents the compartment where leukocytes will have migrated through the tissue microenvironment (i.e. the site of pathology). The concentration of large sCD18 complexes was significantly increased in SF (median 1449 mU/ml, IQR 978–2190 mU/ml) compared with plasma (median 416 mU/ml, IQR 313–588 mU/ml) (Fig. 4B). There was no difference in the concentration of small sCD18 complexes between the two compartments (Fig. 4B lower left panel). To examine whether the concentration of sCD18 complexes reflected the degree of inflammation, the sCD18 concentrations were compared with CRP levels as a systemic marker of inflammation. The concentration of large sCD18 complexes was increased in SF from patients with an elevated CRP (CRP >8 mg/l) (median 2054 mU/ml, IQR 1517–3811 mU/ml) compared with patients with a normal CRP (CRP <8 mg/l) (median 1079 mU/ml, IQR 722–1436 mU/ml) (Fig. 4B). There was no difference in the concentration of small sCD18 complexes when comparing SF from patients with elevated (median 77 mU/ml, IQR 37–134 mU/ml) or normal (median 74, IQR 34–168 mU/ml) CRP (Fig. 4B lower right panel).

## 4 Discussion

Membrane-expressed CD18 integrins are pivotal for cell adhesion and migration. CD18 is shed in response to inflammatory stimuli and migration. The sCD18 forms maintain their ligand binding ability through their association with at least remnant of the CD11 chains, especially CD11a and CD11b, but little was previously known about the function of sCD18 in the immune response. Their ability to compete with the membrane-expressed form of CD18 suggest a role as part of an anti-inflammatory or immunomodulatory mechanisms [[4], [5], [6],^11^], but evidence has been lacking. Here, we present data that supports this hypothesis assigning these properties mainly to the large complexes of sCD18, which are shed when neutrophils migrate across the endothelial barrier. We further introduce ELISA-like assays to estimate the concentrations of the small and large complexes abolishing the need for the more time-consuming procedures involving GPC. The concentration of large complexes are associated with RA suggesting that they are involved in inflammation under pathophysiological conditions.

The concept of sCD18 as an anti-inflammatory agent was previously suggested based on the ability of sCD18 to bind ligands such as ICAM-1 and the association between plasma sCD18 concentrations and disease activity in patients with inflammatory diseases and animal models of inflammatory disease [5,11]. We now show that sCD18 decreased the production of pro-inflammatory cytokine MCP-1 in co-cultures of FLS and PBMC. The effect was similar to the decrease in MCP-1 production seen with disease modifying biological drugs used in the treatment of rheumatoid arthritis [34]. This supports direct anti-inflammatory properties of sCD18 in sites of inflammation, especially in the vessel wall where leukocytes accumulate.

sCD18 complexes are formed mainly in two forms, which can be distinguished by GPC of plasma from both healthy individuals and inflamed patients. Even so, we know little about differences in their production from migrating leukocytes. In the present study, using a SPR-based assay, we found only large sCD18 complexes bound ICAM-1. Furthermore, this binding was dependent on the availability of divalent cations characteristic of activated membrane bound CD18 integrins. By comparing sCD18 complexes from a cell migration assay and an assay of adherent cell contact, we were able to study the timing of CD18 shedding during migration of leukocytes. Neutrophils display a baseline shedding of CD18 that is independent of migration [4] but can be stimulated with activation of adherent cells. However, a marked increase in sCD18 was measured in supernatants from migrated neutrophils as found in the lower compartment of our Boyden-chamber assay. This is in line with previous findings, where cleaving of CD18 was found to be essential for neutrophil detachment from endothelial cells [7]. However, our study adds to the current knowledge in at least three ways. First, it shows the quantitative contributions of baseline, activation-induced and migration-induced CD18 shedding. Furthermore, only large sCD18 complexes were capable of binding ICAM-1. Second, the baseline shedding of CD18 consists primarily of small sCD18 complexes. In contrast, shedding of CD18 resulting from migration of neutrophils produced quantitatively only large sCD18 complexes. Third, neutrophils shed CD18 in the compartment where they end to conclude the transendothelial migration by detaching from the endothelium. This finding immediately suggests a testable hypothesis, namely that large sCD18 complexes are shed at the inflammatory site by neutrophils. Past studies on sCD18 in patients all measured total sCD18 concentrations. We developed a method to estimate the concentrations of specific size subpopulation of sCD18 important for multiple inflammatory markers [35]. Large sCD18 complexes were dramatically increased in SF compared with plasma from patients with RA. This is in line with results from the migration assay, supporting the shedding of large sCD18 complexes at the inflammatory site. Further, the concentration of large sCD18 complexes in SF was strongly associated with CRP in patients with RA, unlike the weaker association, which were reported when total sCD18 was compared against this inflammatory marker [5]. Despite the heterogeneity of this patient group with regard to CRP levels, a strong positive association between CRP and large sCD18 complex levels was found, supporting formation of large sCD18 complexes is associated with high inflammatory activity.

The different functions of sCD18 complexes, especially in relation to their overall size, is an important progress in understanding the impact of these integrins in regulating the inflammatory response. Previously it was assumed that such regulation only related to function of the membrane-bound forms, but our work now opens a perspective, where shed CD18 complexes also are active parts with multiple implications. They modulate secretion by the endothelium of MCP-1, a cytokine with a known chemoattractant property for myeloid leukocytes among other functions [36]. The select binding to ICAM-1 by only large sCD18 complexes and a likewise select shedding of these complexes in inflammatory site adds potential new roles of sCD18 in inflammatory diseases such as analyzed in the present study with a cohort of RA patients. Whether this mechanism is specific to RA or is a more general response to inflammation in other diseases still remains an open question. It is not known what consequences malfunctions in any of the mechanisms pertaining to sCD18 function might have. The expression of ICAM-1 and CD18 integrins is increased in patients with RA, and TNFα induces expression of both ICAM-1 and CD18 as well as activation of CD18 integrins to an active, ligand-binding conformation [[37], [38], [39]]. If sCD18 modulates any of these interconnected mechanisms, defects may well have consequences for disease. Mutation in the *ITGAB* gene encoding the ICAM-1 binding CD11b chain are strongly associated with systemic lupus erythematosus, but so far, an explanation for the functional consequence of such mutation has remained obscure [40]. We suggest that better insight on the structure-function relationship of sCD18 complexes could answer at least some of these questions.

## 5 Conclusion

Taken together, our study now shows sCD18 to play a role in regulation of the immune system, especially relating to leukocyte transmigration through the endothelium. Leukocytes primarily shed large sCD18 complexes when migrating. The large complexes are responsible for the anti-inflammatory properties of sCD18. As a result, sCD18 may hold potential as a biomarker for inflammation autoimmune diseases exemplified here by RA, especially when the size of the complexes is brought into consideration. Furthermore, a mimic of the cytokine-limiting effect exhibited by sCD18 oligomers may open a venue for therapeutic treatment of RA and other inflammatory diseases.

## CRediT authorship contribution statement

**Alexey Ferapontov:** Writing – review & editing, Writing – original draft, Methodology, Investigation, Formal analysis, Data curation, Conceptualization. **Anders Mellemkjær:** Writing – review & editing, Methodology, Investigation, Formal analysis, Data curation, Conceptualization. **Helen M. McGettrick:** Writing – review & editing, Supervision, Methodology, Data curation, Conceptualization. **Thomas Vorup-Jensen:** Writing – review & editing, Writing – original draft, Supervision, Resources, Project administration, Methodology, Funding acquisition, Formal analysis, Conceptualization. **Tue W. Kragstrup:** Writing – review & editing, Writing – original draft, Supervision, Resources, Project administration, Methodology, Funding acquisition, Data curation, Conceptualization. **Kristian Juul-Madsen:** Writing – review & editing, Writing – original draft, Supervision, Project administration, Methodology, Investigation, Formal analysis, Data curation, Conceptualization.

## Availability of supporting data

Please contact corresponding authors for data requests.

## Funding

This work was supported by Independent Research Fund Denmark (9039-00015B), The Lundbeck Foundation (R380-2021-1326), the Faculty of Health at 10.13039/100007605Aarhus University, and The 10.13039/100008368Danish Rheumatism Association.

## Declaration of competing interest

The authors declare the following financial interests/personal relationships which may be considered as potential competing interests:Thomas Vorup-Jensen reports financial support was provided by Independent Research Fund Denmark. Tue W. Kragstrup reports was provided by The Danish Rheumatism Association. Tue W. Kragstrup reports a relationship with Pfizer Inc that includes: speaking and lecture fees. Tue W. Kragstrup reports a relationship with 10.13039/100004312Eli Lilly and Company that includes: consulting or advisory and speaking and lecture fees. Tue W. Kragstrup reports a relationship with Bristol Myers Squibb Co that includes: consulting or advisory and speaking and lecture fees. Tue W. Kragstrup reports a relationship with Novartis that includes: speaking and lecture fees. Tue W. Kragstrup reports a relationship with UCB Inc that includes: consulting or advisory and speaking and lecture fees. Tue W. Kragstrup reports a relationship with AbbVie Inc that includes: speaking and lecture fees. Tue W. Kragstrup reports a relationship with Gilead Sciences Inc that includes: consulting or advisory and speaking and lecture fees. Kristian Juul-Madsen reports financial support provided by the Lundbeck Foundation. If there are other authors, they declare that they have no known competing financial interests or personal relationships that could have appeared to influence the work reported in this paper.

## Data Availability

Data will be made available on request.
