# Supplementary figures and images for: Large soluble CD18 complexes with exclusive ICAM-1-binding properties are shed during immune cell migration in inflammation
Source: J Transl Autoimmun. 2025 Jan 5;10:100266. doi: 10.1016/j.jtauto.2025.100266 (PMC11759538; doi:10.1016/j.jtauto.2025.100266)

Calibration curve ( $M_w$ )

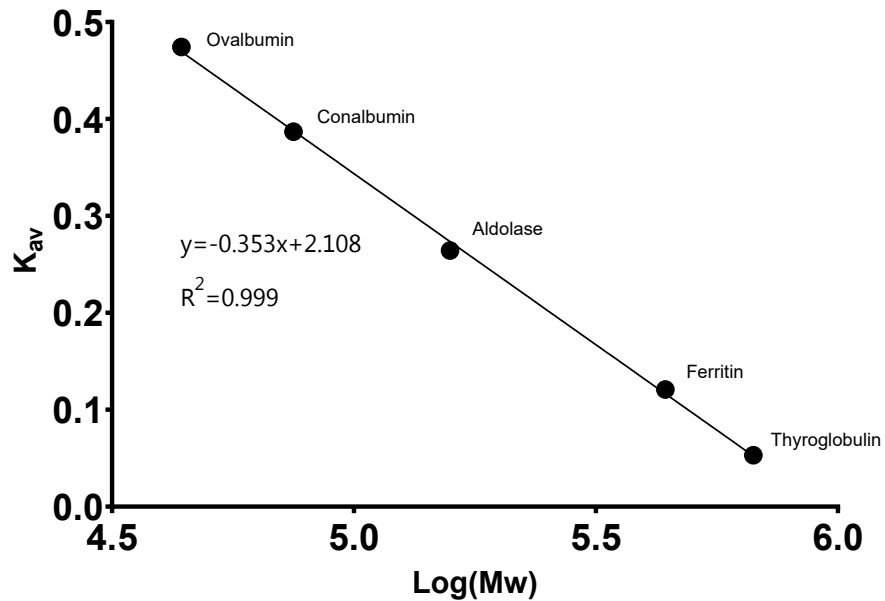

Cs calibration curve (Hydrodynamic radius)

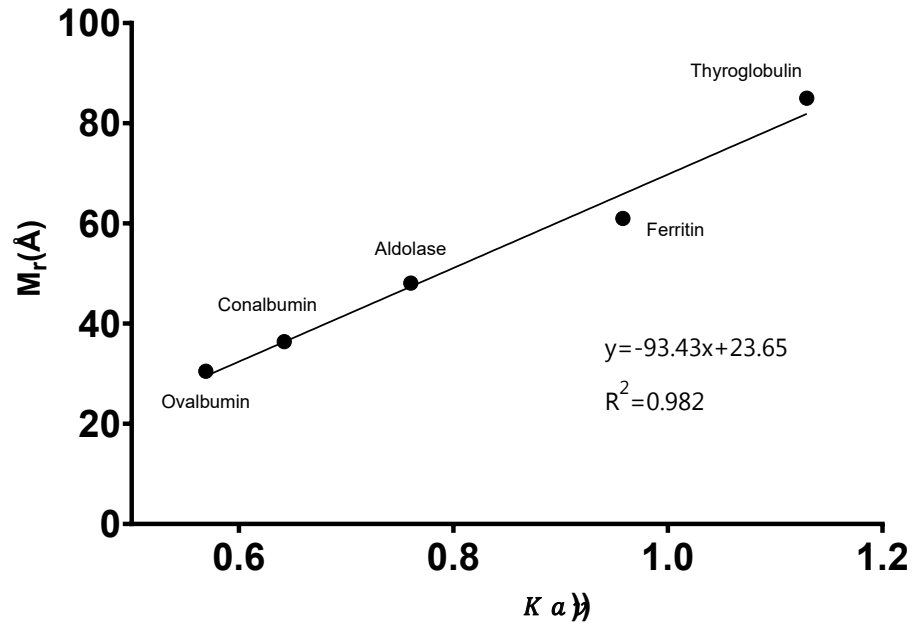

Supplement: Multimedia component 1 [file mmc1.pdf]

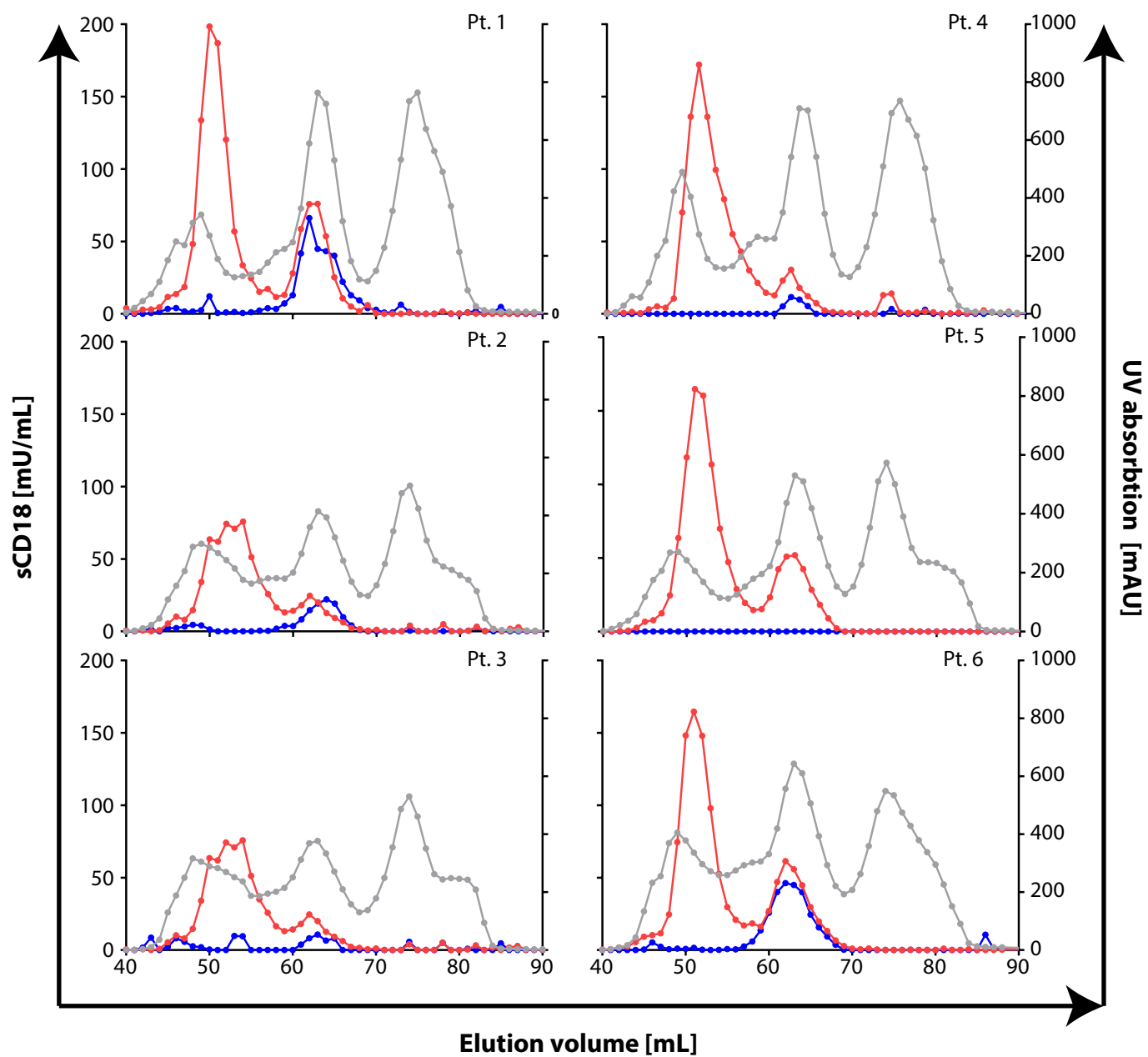

Supplement: Multimedia component 2 [file mmc2.pdf]
